# Supplementary material for: Fostering resilience in conflict-affected schools: A randomized controlled trial of the 3C program’s effects on Afro-Colombian adolescents
Source: Glob Ment Health (Camb). 2025 Dec 17;13:e17. doi: 10.1017/gmh.2025.10119 (PMC12877914; doi:10.1017/gmh.2025.10119)
Supplement: González-Ballesteros et al. supplementary material [file S2054425125101192sup001.docx]

**Supplementary Table S1. Variables with Missing Data at 9-Month Follow-Up and Imputation Methods Used in Multiple Imputation by Chained Equations (MICE).**

| **Scope** | **Construct** | **Type** | **Missing at 9-Month Follow-Up** | **Imputation Method (MICE)** |
| --- | --- | --- | --- | --- |
| Only outcomes with missingness due to 9-month attrition (n = 23 of N = 460; 5.0%) | HARS total (anxiety) | Continuous | 23 cases (5.0%) | Chained linear regression |
|  | PCL-C total (PTSD) | Continuous | 23 cases (5.0%) | Chained linear regression |
|  | CD-RISC (resilience) | Continuous | 23 cases (5.0%) | Chained linear regression |
|  | ECOM (compassion) | Continuous | 23 cases (5.0%) | Chained linear regression |
|  | PSB (prosocial behavior) | Continuous | 23 cases (5.0%) | Chained linear regression |
|  | WDS (depression screen) | Binary | 23 cases (5.0%) | Chained logistic regression |

*Notes*: Missing data were imputed using multiple imputation by chained equations (MICE) in Stata 18 with 23 imputations (m = 23) to account for attrition. Cluster identifiers (school/class) were not imputed but included as predictors in the imputation model.

**Supplementary Table S2. Variables Used in Mixed-Effects Models.**

| **Variable** | **Model Role** | **Type** | **Scale/Possible Values** | **Coding/Reference** | **Transformation** |
| --- | --- | --- | --- | --- | --- |
| CD-RISC (resilience) | Outcome | Continuous | 0–100 | Higher = more resilience | None |
| ECOM (compassion) | Outcome | Continuous | 17–85 | Higher = more compassion | None |
| PSB (prosocial behavior) | Outcome | Continuous | 30–150 | Higher = more prosociality | None |
| HARS total (anxiety) | Outcome | Continuous | 0–56 | Higher = more anxiety | None |
| PCL-C total (PTSD) | Outcome | Continuous | 17–85 | Higher = more PTSD symptoms | None |
| WDS (depression screen) | Outcome (binary model) | Binary | 0 = negative; 1 = positive | Reference = 0 | None |
| Group | Fixed effect | Binary | 0 = control; 1 = intervention | Reference = control | None |
| Time | Fixed effect | Categorical | 0 = baseline; 1 = 6 months; 2 = 9 months | Reference = baseline | None |
| Group×Time | Fixed effect | Interaction | As above | --- | --- |
| Age (years) | Covariate | Continuous | 12–18 | --- | None |
| Gender | Covariate | Binary | 0 = female; 1 = male | Reference = female | None |
| Conflict exposure | Covariate | Binary | 0 = no; 1 = yes | Reference = no | None |
| School | Random effect | Cluster ID | Categorical (levels = schools) | Random intercept | --- |
| Class (nested in school) | Random effect | Cluster ID | Categorical (levels = classes) | Random intercept | --- |

Note: All models included random intercepts at school and class levels to account for clustering. Fixed effects included group, time, group×time interaction, and specified covariates.

**Supplementary Table S3. Sensitivity Analysis Comparing Multiple Imputation and Listwise Deletion for CD-RISC Outcomes.**

| **Outcome** | **Term (vs. baseline)** | **Imputed estimate (β)** | **Listwise deletion estimate (β)** | **Absolute Δ** | **% Δ** | **p (imputed)** | **p (listwise deletion)** | **Conclusion** |
| --- | --- | --- | --- | --- | --- | --- | --- | --- |
| CD-RISC | Group×Time = 6 mo | 13.13 | 13.14 | 0.01 | 0.0007 | <0.001 | <0.001 | Consistent (≤5%) |
|  | Group×Time = 9 mo | 4.68 | 4.69 | 0.01 | 0.002 | 0.002 | 0.002 | Consistent (≤5%) |

*Notes*: Listwise deletion estimates were within ≤5% of imputed estimates for all group×time contrasts, and inferences were unchanged, indicating minimal bias due to attrition.

**Supplementary Table S4. Sensitivity Analysis for CD-RISC Including Baseline Mental Health Covariates.**

| **Variable** | **Coefficient** | **Std. Error** | **t** | **P>t** | **95% Conf. Interval** |
| --- | --- | --- | --- | --- | --- |
| Baseline HARS (anxiety) | -0.154 | 0.070 | -2.20 | 0.028 | [-0.291, -0.017] |
| Baseline PCL-C (PTSD) | 0.068 | 0.047 | 1.44 | 0.151 | [-0.025, 0.161] |
| Group×Time = 6 months | 11.94 | 1.94 | 6.15 | <0.001 | [8.135, 15.751] |
| Group×Time = 9 months | 4.78 | 1.97 | 2.42 | 0.016 | [0.908, 8.647] |
| Age | 0.664 | 0.201 | 3.31 | 0.001 | [0.271, 1.058] |
| Male gender | 1.149 | 2.181 | 0.53 | 0.598 | [-3.125, 5.423] |
| Conflict exposure | 0.708 | 0.934 | 0.76 | 0.448 | [-1.122, 2.538] |

Notes: Model includes random intercepts at school and class levels. Only key variables shown; full model includes group, time, and interaction terms with gender as specified in Methods. Baseline anxiety was negatively associated with resilience (p=0.028), while baseline PTSD showed no significant association. Crucially, intervention effects (Group×Time interactions) remained significant after adjusting for baseline mental health burden, demonstrating that observed resilience gains are not explained by baseline differences between groups.

**Supplementary Table S5. Non-Imputed Measurement Results by Group at Baseline, Endline (6 months), and Follow-up (9 months).**

| **Variable** | **Control**  (N = 174) | **Intervention**  (N = 286) | **Total**  (N = 437) |
| --- | --- | --- | --- |
| **Total anxiety at baseline** | 10.76 (8.08) | 11.50 (7.87) | 11.22 (7.95) |
| **Total anxiety at endline (6 months)** | 11.44 (6.98) | 6.73 (4.86) | 8.51 (6.19) |
| **Total anxiety at follow-up (9 months)** | 10.63 (6.99) | 8.58 (8.22) | 9.37 (7.82) |
| **Baseline anxiety level** | | | |
| Mild | 138 (79.3%) | 220 (76.9%) | 358 (77.8%) |
| Mild to moderate | 24 (13.8%) | 50 (17.5%) | 74 (16.1%) |
| Moderate to severe | 9 (5.2%) | 13 (4.5%) | 22 (4.8%) |
| Very severe | 3 (1.7%) | 3 (1.0%) | 6 (1.3%) |
| **Endline anxiety level** | | | |
| Mild | 142 (81.6%) | 280 (97.9%) | 422 (91.7%) |
| Mild to moderate | 23 (13.2%) | 5 (1.7%) | 28 (6.1%) |
| Moderate to severe | 7 (4.0%) | 1 (0.3%) | 7 (1.5%) |
| Very severe | 2 (1.1%) | 0 (0.0%) | 3 (0.7%) |
| **Follow-up anxiety level** | | | |
| Mild | 146 (86.4%) | 229 (85.4%) | 375 (85.8%) |
| Mild to moderate | 17 (10.1%) | 19 (7.1%) | 36 (8.2%) |
| Moderate to severe | 0 (0.0%) | 11 (4.1%) | 11 (2.5%) |
| Very severe | 6 (3.6%) | 9 (3.4%) | 15 (3.4%) |
| **Baseline depression screening** | | | |
| Negative | 51 (29.3%) | 131 (45.8%) | 182 (39.6%) |
| Positive | 123 (70.7%) | 155 (54.2%) | 278 (60.4%) |
| **Endline depression screening** | | | |
| Negative | 49 (28.2%) | 225 (78.7%) | 274 (59.6%) |
| Positive | 125 (71.8%) | 61 (21.3%) | 186 (40.4%) |
| **Follow-up depression screening** | | | |
| Negative | 42 (24.9%) | 151 (57.4%) | 146 (33.4%) |
| Positive | 127 (75.1%) | 112 (42.6%) | 254 (66.6%) |
| PCL-C Baseline | 33.98 (12.81) | 33.47 (11.21) | 33.66 (11.82) |
| PCL-C Endline | 35.70 (10.67) | 25.11 (7.64) | 28.36 (9.83) |
| PCL-C Follow-up | 34.01 (10.45) | 29.59 (10.06) | 31.30 (10.42) |
| **Baseline PTSD screening** | | | |
| Negative | 78 (44.8%) | 120 (42.0%) | 198 (43.0%) |
| Positive | 96 (55.2%) | 166 (58.0%) | 262 (57.0%) |
| **Endline PTSD screening** | | | |
| Negative | 71 (40.8%) | 234 (81.8%) | 305 (66.3%) |
| Positive | 103 (59.2%) | 52 (18.2%) | 155 (33.7%) |
| **Follow-up PTSD screening** | | | |
| Negative | 64 (36.8%) | 156 (54.5%) | 220 (47.8%) |
| Positive | 110 (63.2%) | 130 (45.5%) | 240 (52.2%) |
| **CD-RISC at baseline** | 65.32 (13.49) | 61.88 (15.91) | 63.18 (15.12) |
| **CD-RISC at endline** | 65.21 (12.06) | 74.91 (10.61) | 71.24 (12.12) |
| **CD-RISC at follow-up** | 65.11 (15.41) | 66.36 (11.05) | 65.88 (12.91) |
| **ECOM at baseline** | 64.56 (11.20) | 62.52 (12.52) | 63.29 (12.07) |
| **ECOM at endline** | 65.09 (9.99) | 67.47 (10.25) | 66.57 (11.37) |
| **ECOM at follow-up** | 64.85 (10.91) | 61.32 (13.70) | 62.68 (12.79) |
| **PSB at baseline** | 85.20 (9.21) | 82.64 (7.69) | 83.60 (8.38) |
| **PSB at endline** | 85.02 (8.37) | 95.17 (7.40) | 91.33 (9.20) |
| **PSB at follow-up** | 84.18 (8.06) | 81.42 (6.33) | 82.49 (7.17) |

*Note:* Total anxiety refers to the global HARS score, while anxiety level refers to the severity indicated by HARS cut-off points. Depression screening was considered positive if at least one of the two WDS questions was answered affirmatively. PTSD screening was assessed using the PCL-C cut-off point. CD-RISC: Connor-Davidson Resilience Scale, ECOM: *Escala de Compasión* (Compassion Scale), PSB: Prosocial Personality Battery.
